# Supplementary material for: Perceptions about and reasons for participation in research bronchoscopy in Uganda: A qualitative analysis
Source: PLoS One. 2023 Oct 20;18(10):e0293174. doi: 10.1371/journal.pone.0293174 (PMC10588838; doi:10.1371/journal.pone.0293174)
Supplement: S1 File — (PDF) [file pone.0293174.s001.pdf]

Pre Bronchoscopy questionnaire for Persons having their First Research Bronchoscopy.

Instructions for interviews:

1. Interview should be in a quiet private area.
2. Ask all of the following questions.
3. Before the interview begins, inform participants that they do not need to answer any questions that they are uncomfortable answering.
4. Record answers in free text during the interview for later categorization for analysis.

Date of interview:

Interviewer:

Participant Identification Number:

Start time:

End time:

BACKGROUND CHARACTERISTICS	
1. How old are you now?	
2. Respondents sex	M<br>F
3. Have you ever been a research participant at this site? If so, when and for how long?	
4. At what age did you start participating in this research?	
5. Did you have any procedures during your earlier research?	
6. What was your relation to the TB case in your household?	
7. In your opinion , how sick was the TB case in your household before starting treatment?	
8. In your opinion what was the outcome of the TB treatment in the TB case	
9. Do you still stay in the same household as the TB case?	1. Y<br>2. N
THE PROCEDURE	
1. Have you ever heard of bronchoscopy before? Please explain	1. Y<br>2. N
2. Why do you think you are being asked to have a bronchoscopy?	
3. Has anyone else in your household been asked to have the bronchoscopy?	

4. What are your feelings regarding the bronchoscopy	
5. Why do you think you feel the way you do about the bronchoscopy	
6. What concerns you about the bronchoscopy?	
7. Do you feel you received enough information to decide about whether or not to have the bronchoscopy	
8. What do you expect from the bronchoscopy?	
9. Do you wish to take part in this research study involving bronchoscopy?	1. Y<br>2. N
10. Why do you wish to take part in this research?	
11. Why don't you wish to take part in this study?	
12. What do you expect from the bronchoscopy?	
