# Supplementary material for: Perceptions about and reasons for participation in research bronchoscopy in Uganda: A qualitative analysis
Source: PLoS One. 2023 Oct 20;18(10):e0293174. doi: 10.1371/journal.pone.0293174 (PMC10588838; doi:10.1371/journal.pone.0293174)
Supplement: S2 File — (PDF) [file pone.0293174.s002.pdf]

Post-Bronchoscopy quetionnaire for Persons Who Have Had A Research Bronchoscopy

Instructions for interviews:

1. Interview should be in a quiet private area.
2. Ask all of the following questions.
3. Before the interview begins, inform participants that they do not need to answer any questions that they are uncomfortable answering.
4. Record answers in free text during the interview for later categorization for analysis.

Date of interview:

Interviewer:

Participant Identification Number:

Start time:

End time:

1. How was your experience with your recent bronchoscopy?

2. Tell me about any problems you had during or after bronchoscopy.

3. What advice would you give to someone who is going to have a research bronchoscopy?

4. Is there anything you would like to have changed about the bronchoscopy or your care afterwards?

5. How did the information you received before the bronchoscopy affect you?

6. Did you have an opportunity to ask questions about bronchoscopy before you had it?

7. Do you feel that you got enough information from the study staff about bronchoscopy?

8. Why did you take part in this research study?

9. Would you be willing to participate in another research bronchoscopy in the future?
