# Supplementary material for: Perceptions about and reasons for participation in research bronchoscopy in Uganda: A qualitative analysis
Source: PLoS One. 2023 Oct 20;18(10):e0293174. doi: 10.1371/journal.pone.0293174 (PMC10588838; doi:10.1371/journal.pone.0293174)
Supplement: Attachment — Submitted filename: Response to Reviewers.pdf [file pone.0293174.s003.pdf]

PONE-D-23-01054
Perceptions about and reasons for participation in research bronchoscopy in Uganda: a qualitative analysis
PLOS ONE

Response to Reviewer Comments

Review Comments to the Author
Reviewer #1:
Comment:
1. A reflexivity statement from the authors explaining who they are, their background, and how this shapes both the questions asked and the likely way in which the interviews generated data. This needs contextualising in Uganda and the way people engage with hierarchy and research activities. I think the key to interpreting qualitative data is in understanding any potential biases or confounding factors in the data - why might the research participants chosen give the particular answers they did to these researchers at this time and in this context? Might an alternative study find different accounts (for example if interviewing them in their home setting, or in a group?) and why might these accounts vary? Would a different research team find different results because of the nature of the interviewers?
Response:
We have expanded discussion of the issues of reflexivity and potential bias to the findings due to the research context in the introduction (lines 98-100) and the study limitations section (lines 365-371). The Uganda-CWRU Research Collaboration's (UCRC) TB Project Clinic has been in operation in this area for over 30 years. As such the chances that the study participants or their household members have previously heard about or has prior contact with health care at the clinic are high. All of the participants were household contacts of index TB patients who received TB treatment from the UCRC clinic. They may have experienced some exposure to the UCRC research and clinic operations. However we strongly believe that given the trained social scientists and study procedures to gain consent that the responses were not biased to a point where they may negatively impact the conclusions.

Comment:
2. The details of the analysis are too thin for me at the moment. All the right work has been done, but I need to better understand the a priori codes and where they came from, how the literature was reviewed, what research team experience went into them etc. The a priori codes are so important as they expose the underlying logic model of the research team, the results they might 'expect' to find and the existing themes within the literature. The literature review methods is also essential - was this systematic or narrative, and how might any biases in it inform these a priori codes? Following this, I think it's important to see a bit more of the code book, the themes that began and then emerged, and how they were grouped and/or abandoned to come up with the final themes.
Response:
Thank you for the comments on better understanding the coding process. We conducted a detailed review of the limited medical literature about perceptions and acceptability of

research bronchoscopy in clinical research in low and middle income countries including correspondence and sharing of experience with investigators in Malawi and Norway who have worked in this area. We have added additional description (lines 156-160) explaining how we selected some codes a priori – from literature on the subject which we reviewed specifically from prior knowledge. We have attached our code books with this resubmission to illustrate how we reached the final themes. An extract of the codebooks could be included as supplementary material in the publication. We feel that the comment and explanations have strengthened the analysis section.

Comment:
3. Developing this point, I think a clear understanding of the analysis technique would help. there are now so many variations on reflexive thematic analysis and it can be useful to understand which variant was chosen and why. If theme generation is they key, these themes need to be clearly listed and defended (sometimes a rich infographic is best for these to try to show how they interact with each other and with any overarching or meta themes).
Response:
We have expand discussion on the analytical process (lines 143-146) as described above and included additional references (see below and references 9-10 on line 144) supporting and explaining the approach in more detail. We agree that theme generation was an important part of the analysis and has an influence on how we draw our conclusions.

The additional references are:

Braun, V., Clarke, V.: Thematic analysis. In: Cooper, H., Camic, P.M., Long, D.L., Panter, A.T., Rindskopf, D., Sher, K.J. (eds.) APA Handbook of Research Methods in Psychology, Research Designs, vol. 2, pp. 57–71. American Psychological Association, Washington (2012)

Braun, V., Clarke, V.: Reflecting on reflexive thematic analysis. Qual. Res. Sport Exerc. Health 11(4), 589–597 (2019). https:// doi. org/ 10. 1080/ 21596 76X. 2019. 16288 06

Comment:
4. These themes then need to pull through more strongly into the results and discussion. At the moment, the statements are quite weak 'some participants responded...' and even where the number is quoted such as n=12 this does not really convey how strongly these points were made in the data. I also felt the need to really understand more what people said (I suspect no respondent actually used the words 'therapeutic misconception' so it is really important to see how this term emerged from their lived experiences). The quotes used are excellent but a little sparse. Could there be more in accompanying data if space does not permit longer quotes in the body text?
Response:
We have revised the results and discussion to strengthen them and discuss the lived experiences in more depth. We have added additional vignettes to the paper to provide more insight about therapeutic misconception. The discussion now includes the important

point about blurred lines between actual, plausible intent to gain some health care benefits as the UCRC clinic and the misperception that undergoing bronchoscopy as part of research was likely to lead to TB treatment. The additional text can be found in the Results (lines 178-189; 202-239) and the Discussion (lines 329-340; 356-361; 365-371).

Comment:
5. The core notion of therapeutic misconception I felt needed more unpacking. To the newcomer, this sounds like people thinking they are going to get treatment which they are not. However it seems to overlap with people using research participation to understand more about their own health, which is not seemingly the same thing. Having a load of blood tests and a bronco definitely will tell you something about your health, even if not therapeutic - I wonder where these boundaries lie. Did some participants really think they were going to get better as a result of the branch study? Presumably in the ethics for the study, and health issue which is identified must then also be treated or referred - so in essence maybe there is a valid therapeutic element to participating? Is this then a misconception or simply a different perspective?

Trying to bring these points together, I feel as though there needs to be some revision to help the interested reader get closer to the lived experience of the participants, and then understand the inferences drawn by the research team to synthesise these into generalisable concepts. This mostly involves a more detailed account of the data creation and analysis to show how the data was obtained and processed to create these insights, but once done will prompt a slightly revised discussion section.

For all this, I reiterate that I think the paper is an excellent concept and am sure the data obtained is of a high quality - I just to need to be shown more of it!
Response:
We agree with both comments 4 and 5 above and have revised and expanded the Results and Discussion. The additional text discussing therapeutic misconception can be found in the Results (lines 178-189; 202-239) and Discussion (lines 329-340; 356-361; 365-371). We have now brought the reader closer to the lived experiences by adding more quotes from the data. We have also attached the codebooks which may be provided as supplementary material for the reader to further engage with the way we engaged with the lived experiences.

Journal Requirements
When submitting your revision, we need you to address these additional requirements.
Comment:
1. Please ensure that your manuscript meets PLOS ONE's style requirements, including those for file naming. The PLOS ONE style templates can be found at https://journals.plos.org/plosone/s/file?id=wjVg/PLOSOne_formatting_sample_main_body.pdf and https://journals.plos.org/plosone/s/file?id=ba62/PLOSOne_formatting_sample_title_authors_affiliations.pdf

Response:
The manuscript now meets PLOS ONE's style requirements, including those for file naming.

Comment:
2. Thank you for stating the following financial disclosure:
"The funders had no role in study design, data collection and analysis, decision to publish, or preparation of the manuscript."
At this time, please address the following queries:
a) Please clarify the sources of funding (financial or material support) for your study. List the grants or organizations that supported your study, including funding received from your institution.
b) State what role the funders took in the study. If the funders had no role in your study, please state: "The funders had no role in study design, data collection and analysis, decision to publish, or preparation of the manuscript."
c) If any authors received a salary from any of your funders, please state which authors and which funders.
d) If you did not receive any funding for this study, please state: "The authors received no specific funding for this work."
Please include your amended statements within your cover letter; we will change the online submission form on your behalf.
Response:
We have amended the funding statements provided in the first submission and added information that was originally stated in the acknowledgements section yet it should have appeared in the funding statement. The new funding statement reads as follows:
"The parent study was funded by grants and contracts UO1AI115642, RO1AI124348 and 75N93019C00071 from the U.S. National Institutes of Health. The funders had no role in study design, data collection and analysis, decision to publish, or preparation of the manuscript."

Comment:
3. Thank you for stating the following in the Acknowledgments Section of your manuscript:
"The parent study was funded by grants and contracts UO1AI115642, RO1AI124348 and 75N93019C00071 from the U.S. National Institutes of Health."
We note that you have provided additional information within the Acknowledgements Section that is not currently declared in your Funding Statement. Please note that funding information should not appear in the Acknowledgments section or other areas of your manuscript. We will only publish funding information present in the Funding Statement section of the online submission form.
Please remove any funding-related text from the manuscript and let us know how you would like to update your Funding Statement. Currently, your Funding Statement reads as follows:
"The funders had no role in study design, data collection and analysis, decision to publish, or preparation of the manuscript."

Please include your amended statements within your cover letter; we will change the online submission form on your behalf.

Response:

We have amended the Acknowledgement section by removing funding-related text from the manuscript (lines 378-380). The new acknowledgement text reads as follows:

"We thank the participants who generously gave us their time and attention to complete this study."

Comment:

4. In your Data Availability statement, you have not specified where the minimal data set underlying the results described in your manuscript can be found. PLOS defines a study's minimal data set as the underlying data used to reach the conclusions drawn in the manuscript and any additional data required to replicate the reported study findings in their entirety. All PLOS journals require that the minimal data set be made fully available. For more information about our data policy, please see http://journals.plos.org/plosone/s/data-availability.

Upon re-submitting your revised manuscript, please upload your study's minimal underlying data set as either Supporting Information files or to a stable, public repository and include the relevant URLs, DOIs, or accession numbers within your revised cover letter. For a list of acceptable repositories, please see http://journals.plos.org/plosone/s/data-availability#loc-recommended-repositories. Any potentially identifying patient information must be fully anonymized.

Important: If there are ethical or legal restrictions to sharing your data publicly, please explain these restrictions in detail. Please see our guidelines for more information on what we consider unacceptable restrictions to publicly sharing data: http://journals.plos.org/plosone/s/data-availability#loc-unacceptable-data-access-restrictions. Note that it is not acceptable for the authors to be the sole named individuals responsible for ensuring data access.

We will update your Data Availability statement to reflect the information you provide in your cover letter.

Response:

The minimal, de-identified data set underlying the results of this manuscript is located on a local server at the Uganda-Case Western Reserve University Collaboration Tuberculosis Research Project Clinic's data center. It is now available through email (data@mucwru.or.ug) upon reasonable request, with permission and notification of the Makerere University School of Social Sciences Research Ethics Committee (MAKSS REC). Any potentially identifying patient information has been fully anonymized.

Comment:

5. We note that you have stated that you will provide repository information for your data at acceptance. Should your manuscript be accepted for publication, we will hold it until you provide the relevant accession numbers or DOIs necessary to access your data. If you wish to make changes to your Data Availability statement, please describe these changes in your cover letter and we will update your Data Availability statement to reflect the information you provide.

Response:
The minimal, de-identified data set underlying the results of this manuscript is located on a local server at the Uganda-Case Western Reserve University Collaboration Tuberculosis Research Project Clinic's data center. It is now available through email (data@mucwru.or.ug) upon reasonable request, with permission and notification of the Makerere University School of Social Sciences Research Ethics Committee (MAKSS REC). Any potentially identifying patient information has been fully anonymized.

Comment:
6. Please include your tables as part of your main manuscript and remove the individual files. Please note that supplementary tables (should remain/ be uploaded) as separate "supporting information" files.
Response:
We have included our table as part of the main manuscript (lines 170-171) and removed the individual file. We have included extracts of the codebook as separate "supporting information" files under supplementary material.
