# Supplementary material for: Perceptions about and reasons for participation in research bronchoscopy in Uganda: A qualitative analysis
Source: PLoS One. 2023 Oct 20;18(10):e0293174. doi: 10.1371/journal.pone.0293174 (PMC10588838; doi:10.1371/journal.pone.0293174)
Supplement: Attachment — Submitted filename: Point by Point Response to Reviewers_02082023.pdf [file pone.0293174.s004.pdf]

PONE-D-23-01054R1
Perceptions about and reasons for participation in research bronchoscopy in Uganda: a qualitative analysis
PLOS ONE

Response to Reviewer Comments

Review Comments to the Author
Reviewer #1:
Comment:
Thanks for these careful amendments in response to my original review - they go a long way to addressing my questions. I have only minor comments in response.

1. 'A reflexivity statement from the authors'. I think my original point has been slightly missed. I have faith in the expertise of the authors and their study protocol, and do not question their conclusions. However all qualitative research has a degree of subjectivity and inherent bias, which I think needs more reflection and understanding when presenting the findings. Importantly, this does not weaken the conclusions, in my view. As the authors note, their collaboration has been running a long time and there will be a great degree of knowledge of it (as indeed comes across in the quotes). Given this, and the position of the researchers as affiliated to the programme, how might this have focussed the responses given by the participants? I would expect any person in any context to have a range of complex motivations for a given action (some implicit, some examined), underpinned by a biopsychosocial narrative about their health. What such narratives exist in Uganda? What might be the role of faith, health beliefs, and community pressure and which of these might not be volunteered to academic researchers affiliated to an established health outreach programme? The authors also started with a code book drawn from literature (as opposed to say a Community Engagement exercise) - how does this direct the interpretation of the data generated? I think it's important to identify a grounding in a given model of health, disease, and culture so that the findings are grounded in a given perspective. The few lines added do not quite address this for me and I am intrigued (for my own interest as well as academic rigour) to read what the author perceptions are as to what things they were told but also might not have been told, and why the respondents might have given that particular account at that particular time. Is it possible to see the prompting questions for the interviews?

Response:
Thank you for this additional clarification on the earlier comment. We appreciate the need to provide more insight and discussion about the issue of reflexivity. We note that in fact our position in the program may influence our interpretation of the participants' views just as their responses may be influenced by the background context of their engagement with UCRC's research and treatment agenda. As mentioned earlier, the UCRC research and associated health care services has been ongoing for over 30 years in this study context. Moreover, the authors as well as the study staff have also had two or more decades of familiarity with research in the study area. We recognize that our interpretations of participants' reasons for willingness to participate in research

bronchoscopy may be influenced by our position as longstanding researchers in this area. For instance, it is possible that our understanding and interpretation of some of the narratives about willingness to participate may be borne out of our bias as longstanding researchers in the area. However, we are confident in our methodological rigor such as ensuring that we used independent, trained social science research assistants to conduct the interviews. This was done in part to improve on internal validity of the interview process. We still acknowledge that for participants, there is a possibility that the association with the UCRC broader research and treatment agenda may have overridden the efforts to ensure they answer honestly about their perceptions of research bronchoscopy.

Our study is potentially influenced by adaptations of the health belief model (HBM):

> Rosenstock IM, Strecher VJ, Becker MH. Social learning theory and the Health Belief Model. Health Educ Q. 1988 Summer;15(2):175-83. doi: 10.1177/109019818801500203.
>
> Abraham, C., & Sheeran, P. (2015). The health belief model. Predicting health behaviour: Research and practice with social cognition models, 2, 30-55).

The HBM posits that individuals will seek care once the perceived risk to their health supersedes other alternatives, mainly 'riding it out' self-medication or treatment. Because some of the participants indicated that their willingness was influenced in part due to their prior experience seeing loved ones being treated well, and the gravity of TB, it is possible that they felt a need to also participate in efforts associated with TB control. Research bronchoscopy in this case would have been perceived in that lens, and so participants may have selected to undergo the procedure having perceived TB as so severe not to engage with care. Part of this explanation is now included in the manuscript on lines 156-160 in the analysis section and 377-384 in the discussion section.

We have attached the interview guide here for the reviewer to see the anticipated probes (prompting questions).

Comment:
2 & 3. 'The details of the analysis'. This is well addressed, but I still do not see a clear delineation of the final codes and themes identified at the completion of the rTA? If a thematic analysis has been performmed, can the authors say clearly what their final themes are and how they are linked?

Response:
We have added additional explanation to link the main themes to the codes (see lines 180-187 in the findings section). The major themes that emerged for willingness to participate in research bronchoscopy as derived from reasons for participation were prior experience of TB having witnessed the suffering and healing process of loved ones as they underwent treatment; a need to 'know their health status' given the range of tests and other care procedures associated or anticipated with being a participant on the study; and therapeutic misconception about gaining treatment despite being healthy adults without TB. An important theme was willingness to participate for altruistic reasons

indicating an appreciation of how research may help generate important knowledge to improve care for future patients.

Comment:
4. 'These themes then need to pull through more strongly into the results and discussion'. The addition of more quotes really helps, thanks for this. I could still use some clearer signposting as to how the quotes chosen link directly to very clear thematic findings, but they very well articulate the broad discussion areas which are worked through.

Response:
We have added more clarity in the findings and discussion about the main themes as discussed above. Some additional subheadings and signposting has been added to link the findings to the discussion.

Comment:
5. 'The core notion of therapeutic misconception.' This was much better in this version, and I felt like I got the heart of it. The extra quotes again really helped. Annoyingly I could not see the attached codebooks on Editorial Manager which I'm sure is a software issue rather than the authors' fault!
Response:
Thank you for appreciating the additional clarity to explain the notion of therapeutic misconception. We have re-attached the codebooks and hope they will be visible/accessible.

Comment:
In summary I think this remains a great paper. I still feel the need for more critical insight into the authors' views as to why they as a research team got this data, and what data they might have missed or chosen not to elicit. I'm still left with a slightly vague feeling as to the key themes the team feel they want to highlight, and how they feel these themes might be linked in a casual or narrative model. However these things are increasingly minor points, driven by a reviewer who is interested in both the subject matter and the methodology!
Response:
Thank you for this helpful comment. We were motivated to undertake this study because of the growing use of bronchoscopy in both research and treatment contexts. But we were unaware what the community of participants in our catchment area appreciate about the procedure as used for research in healthy individuals. We needed to confirm some of the reasons for willingness to participate in an invasive procedure for research purposes when they are healthy adults without TB. All the information will help us to further improve the counseling and education sessions our investigators and staff have with research participants. We have discussed these points further in the discussion and added additional text to clarify these themes in the revised manuscript (see lines 363-384).

Reviewer #2:
Comment:
I am not too sure if I quite approve of this study. This is a research study done on healthy

subjects and the authors have gone for an invasive procedure like bronchoscopy which could have been bypassed for non invasive or less invasive procedures maybe.The authors themselves have said there were lots of apprehensions regarding the procedure. Could they give more explicit details regarding whether they didn't consider it necessary for a bystander to be accompanying the subject when they were doing an invasive procedure? If in case, there would have been complications in the event of bystanders not being there, how would they have dealt with it? How many subjects did not have bystanders accompanying them for the procedure? Was this point not raised by the IEC? This being a small study and going by what the authors say that it was done in a research sensitised area, the results cannot be generalised.

Response:

Thank you for these important observations. Fiberoptic bronchoscopy with bronchoalveolar lavage is a widely used safe method that has been used to study lung diseases such as asthma, interstitial lung disease, sarcoidosis and tuberculosis over the past 40 years. Clinical research units in South Africa and Malawi also perform research bronchoscopy with bronchoalveolar lavage in many of their studies. The main mode of transmission of tuberculosis in humans is inhalation of infectious aerosol droplet nuclei containing small numbers of viable tubercle bacilli. Initial interactions with immune cells in the lung are likely to be important and can not be studied in blood samples. The main objective of the parent study for this substudy of participant acceptability and perceptions about research bronchoscopy in healthy adults in Uganda is to study immunological responses to TB infection in the lung and blood to understand host responses to TB infection and inform the development of safer and more effective TB vaccines, a major global public health need. The parent study is sponsored by the US National Institutes of Health and has undergone full scientific and ethical review and approval in Uganda and the U.S. Two local independent medical safety monitors, who are chest physicians, monitor the study. Adverse events have been infrequent and have consisted mainly of minor self-limited sore throat and cough.

Consent for research bronchoscopy is obtained by trained nurse counselors and the investigators. Participants also receive an IRB-approved information sheet about research bronchoscopy. Participant's have multiple opportunities to ask questions and have them answered before bronchoscopy. Participants are free to withdraw from the study at any time.

Participants undergo full medical evaluation before research bronchoscopy. Research bronchoscopy is done by experienced chest physicians assisted by trained nurses with an anesthetist on standby. Participants undergo monitoring of their their pulse, blood pressure and blood oxygen saturation during bronchoscopy and are observed by medical staff for one hour after the procedure and seen in follow-up 48 to 72 hours later.

Additional Editor Comments

Comment:

Would you please respond to the comments raised by the editors and please submit the full data compilation associated with this study?

Response:
We have responded to the comments raised by the editors and submitted full data compilation for this study.
