# Supplementary material for: Perceptions about and reasons for participation in research bronchoscopy in Uganda: A qualitative analysis
Source: PLoS One. 2023 Oct 20;18(10):e0293174. doi: 10.1371/journal.pone.0293174 (PMC10588838; doi:10.1371/journal.pone.0293174)
Supplement: Attachment — Submitted filename: Response to Reviewers_02102023.pdf [file pone.0293174.s005.pdf]

PONE-D-23-01054R3
Perceptions about and reasons for participation in research bronchoscopy in Uganda: a qualitative analysis
PLOS ONE

Response to Reviewer Comments

Associate Editor's comments:
Comment:
1. We note that your manuscript is not formatted using one of PLOS ONE's accepted file types. Please reattach your manuscript as one of the following file types: .doc, .docx, .rtf, or .tex (accompanied by a .pdf).
If your submission was prepared in LaTex, please submit your manuscript file in PDF format and attach your .tex file as "other."

Response:
Thank you for the comment. We have now attached the manuscript following file types: .doc (accompanied by a .pdf).

Comment:
2. Your ethics statement should only appear in the Methods section of your manuscript. If your ethics statement is written in any section besides the Methods, please move it to the Methods section and delete it from any other section. Please ensure that your ethics statement is included in your manuscript, as the ethics statement entered into the online submission form will not be published alongside your manuscript.

Response:
Our ethics statement now only appears in the Methods section of the manuscript. It is placed at the end of the methods section just before the findings.
